# Antimicrobial Properties of a Peptide Derived from the Male Fertility Factor kl2 Protein of *Drosophila melanogaster*

**DOI:** 10.3390/cimb44030076

**Published:** 2022-02-28

**Authors:** Bernadetta Bilska, Urszula Godlewska, Milena Damulewicz, Krzysztof Murzyn, Mateusz Kwitniewski, Joanna Cichy, Elżbieta Pyza

**Affiliations:** 1 Department of Cell Biology and Imaging, Institute of Zoology and Biomedical Research, Jagiellonian University, 30-387 Cracow, Poland; bernadetta.bilska@doctoral.uj.edu.pl (B.B.); milena.damulewicz@uj.edu.pl (M.D.); 2 Department of Immunology, Faculty of Biochemistry, Biophysics and Biotechnology, Jagiellonian University, 30-387 Cracow, Poland; urszula.m.godlewska@gmail.com (U.G.); mateusz.kwitniewski@uj.edu.pl (M.K.); joanna.cichy@uj.edu.pl (J.C.); 3 Laboratory of Host-Microbiome Interactions, Nencki Institute of Experimental Biology, 02-093 Warsaw, Poland; 4 Department of Computational Biophysics and Bioinformatics, Faculty of Biochemistry, Biophysics and Biotechnology, Jagiellonian University, 30-387 Cracow, Poland; krzysztof.murzyn@uj.edu.pl

**Keywords:** antimicrobial proteins, p4 peptide, *Drosophila melanogaster*, circadian rhythms

## Abstract

Antimicrobial peptides (AMPs) are important components of innate immunity. Here, we report the antimicrobial properties of a peptide derived from the Male fertility factor kl2 (MFF-kl2) protein of *Drosophila melanogaster*, which was identified as a functional analog of the mammalian antibacterial chemerin-p4 peptide. The antimicrobial activity of multifunctional chemerin is mainly associated with a domain localized in the middle of the chemerin sequence, Val66-Pro85 peptide (chemerin-p4). Using bioinformatic tools, we found homologs of the chemerin-p4 peptide in the proteome of *D. melanogaster*. One of them is MFF-p1, which is a part of the MFF kl2 protein, encoded by the gene *male fertility factor kl2* (*kl-2*) located on the long arm of the Y chromosome. The second detected peptide (Z-p1) is a part of the Zizimin protein belonging to DOCK family, which is involved in cellular signaling processes. After testing the antimicrobial properties of both peptides, we found that only MFF-p1 possesses these properties. Here, we demonstrate its antimicrobial potential both in vitro and in vivo after infecting *D. melanogaster* with bacteria. MFF-p1 strongly inhibits the viable counts of *E. coli* and *B. subtilis* after 2 h of treatment and disrupts bacterial cells. The expression of *kl-2* is regulated by exposure to bacteria and by the circadian clock.

## 1. Introduction

Antimicrobial peptides (AMPs), also known as “host-defense peptides”, exhibit antibacterial, antifungal, and antiviral potency. They are highly diverse within and across species, wherein more than 3100 various AMPs have been described from different plant and animal species [1]. Because of the absence of adaptive immune responses in insects, AMPs are essential for insect innate immunity in fighting pathogens [2]. They are intensively studied due to their potential applications as novel antimicrobial drugs [3] and surface antiseptics [4]. So far, seven well-characterized families of inducible AMPs have been identified in *Drosophila melanogaster*. These include: antibacterial Diptericins, Drosocins and Attacins [5,6,7,8,9], antifungal Drosomycins and Metchnikowin [10,11], and Cecropins and Defensin, which exhibit both antibacterial and antifungal activities [12,13,14,15]. The antimicrobial properties of these AMPs have been demonstrated in in vitro studies or have been predicted by in silico analysis.

One of the newly characterized AMPs in humans and rodents is chemerin, a chemoattractant and adipokine [16]. Chemerin is broadly expressed, for example, in environmentally exposed barrier organs, where due to its antimicrobial and chemotactic properties, it likely plays a role in restricting potential microbial invasion [17,18,19]. In human keratinocytes, chemerin is upregulated by exposure to bacteria and acute-phase cytokines [20]. Human chemerin-derived peptide p4 located in the central region of the chemerin sequence is responsible for the antibacterial activity of this protein [16] against a broad range of microorganisms, including skin-, lung-, and oral cavity-associated bacteria [21,22,23]. In a previous study, it was shown that the shorter peptide within p4, chemerin-8 (VR-15), which corresponds to the sequence Val 66-Arg 80 of chemerin, is responsible for the antimicrobial activity of p4 [24]. To better understand the relevance of chemerin-derived antimicrobial peptides in controlling microbial burden, we used *D. melanogaster* as a model when looking for peptides that share similar sequence homology and biochemical features with the antimicrobial peptides chemerin-p4 and chemerin-8 (VR-15).

The main goal of this work was to identify sequence homologs and functional analogs of antibacterial chemerin-derived peptides in the proteome of *D. melanogaster*. We used bioinformatics tools to scan the non-redundant sequence database of *D. melanogaster* proteins, imposing the search criteria related to chemerin-p4 antibacterial activity. We validated the results of our in silico predictions by describing in detail the effects of treatment of various bacterial strains with newly identified *D. melanogaster* peptides. We also assessed how these peptides affect bacterial morphology. Additionally, we investigated whether the expression of the *D. melanogaster* gene, encompassing one of the identified peptides, oscillates daily and if it is modulated by bacterial exposure. Finally, we verified its antimicrobial potential in vivo using transgenic flies with the silenced gene which encodes the tested peptide.

## 2. Materials and Methods

### 2.1. Fly Strains

The following strains of *Drosophila melanogaster* were used for experiments: wild-type *Canton S* (CS), null mutant of the clock gene *period* (*per^01^)*, *white^1118^* (a kind gift from Dr. François Rouyer, Paris Saclay Institute of Neuroscience), UAS-*Valium10* (BDSC, stock no. 35786), UAS-*kl-2^RNAi^* (VDRC, stock No. v19181), and *actin*-GAL4 (a kind gift from Dr. David Dolezel, České Budějovice Biology Centre CAS). Flies were maintained on a standard cornmeal medium under light/dark conditions of LD12:12 (12 h of light and 12 h of darkness) or constant darkness (DD) at 25 °C. 

In all experiments, 7–8-day-old males were used. The progeny of UAS-*kl-2^RNAi^* and *white^1118^*, UAS-*kl-2^RNAi^* and UAS-*Valium10*, and *actin*-GAL4 and *white^1118^* were used as controls for *act* > *kl-2* flies. 

To define the rhythm of *kl-2* expression in *D. melanogaster*, we used *Canton S* and *per^01^* 7–8-day-old males. Following their eclosion, experimental flies were kept in LD 12:12 for 5–7 days and sacrificed in the middle of the day at ZT4 (four hours after lights on) and in the middle of the night at ZT16 (four hours after lights off), or, after 4 days in LD 12:12, they were transferred to DD for a further 2 days. On the third day of DD, they were sacrificed at CT4 or CT16 (CT0—beginning of the subjective day; CT12—beginning of the subjective night). Flies were examined at the aforementioned time points, based on our previously published results [25], and high activity in the immune system of *Drosophila* was shown at ZT16. In total, 30 males were used for a single sample. The abdomens were homogenized in TRIzol (Zymoresearch) and kept at −80 °C until further processing.

### 2.2. Bacterial Strains

The bacterial strains used in the study were *Escherichia coli* HB101, a conventional laboratory strain, WT *Staphylococcus aureus* 8325-4, and *Bacillus subtilis* ATCC 9372.

### 2.3. Peptides

The chemerin-p4 and *Drosophila melanogaster* MFF-p1 and Z-p1 peptides were chemically synthesized by Caslo (Konges Lyngby, Denmark) at 99.98% purity.

### 2.4. Sequence Analysis

To scan a non-redundant peptide sequence database for chemerin peptide analogs, the PHI-BLAST program [26] with default options and limits imposed on the search scope of *Drosophila melanogaster* (NCBI TaxID: 7227) peptide sequences was used. The query sequence was either chemerin-p4 or chemerin-8 (VR-15), with the appropriate RE patterns following those registered in the patent PL 236 566. The RE patterns ensured that the *Drosophila* peptides had amino acid residues in a peptide sequence, characteristic of the chemerin-p4 or chemerin-8 (VR-15) peptides. A search hit was considered significant if the aligned database sequence was cationic at neutral pH and the appropriate peptide showed the marked amphipathicity, assuming either the fully α-helical or fully extended conformation, as indicated by the elevated values of the corresponding relative hydrophobic moments (rHMα and rHMβ, respectively) [27]. Peptide secondary structure predictions were performed using the JPRED4 web server [28]. The JPRED4 server requires the length of a peptide to be at least 30 amino acid long to determine the secondary structure. Thus, in our predictions, we used peptide sequences that were extended at both termini so that the fragments aligned to the appropriate chemerin peptides were flanked for at least five amino acid residues on either side. It was found that changes in a flanking fragment length (ranging from five to ten residues) did not affect the secondary structure prediction results.

### 2.5. Antimicrobial Assays

Bacteria were grown in brain heart infusion (BHI) medium broth at 37 °C. For the microdilution assay (MDA) and minimal inhibitory concentration (MIC) assay, bacteria in the mid-logarithmic phase were harvested and diluted to 4 × 10^5^ CFU/mL with Dulbecco’s PBS. Bacteria were incubated with a series of 2-fold dilutions of indicated peptides or PBS for 2 h. The number of viable bacteria were enumerated by counting the colony-forming units (CFU). The MIC was defined as the lowest antimicrobial concentration which prevents the visible growth of bacteria. 

### 2.6. Drosophila Infection

Based on our previous studies, the middle of the night (ZT16 (Zeitgeber time), 4 h after lights off)) in LD12:12 was selected as the time point to carry out experiments (the maximum expression of *kl-2* in the abdomen) [25]. Flies were anesthetized at ZT16 and then pricked in the thorax with a needle that was previously dipped into a concentrated bacterial solution of *E. coli*. A pure PBS injection was used as a negative control. Injected flies were kept at 25 °C for further analyses.

### 2.7. qRT-PCR

Canton S males were inoculated in the thorax with *E. coli*, PBS or left unmanipulated. Following injection, the flies were placed in vials and left at 25 °C for 3, 6 or 9 h. Next, groups of 15 flies were homogenized in TRIzol (Zymoresearch) and stored at −80 °C until further processing. RNA was isolated from whole flies or abdomens, respectively, using the standard TRIzol extraction procedure. cDNA synthesis was carried out using a High-Capacity cDNA Reverse Transcription Kit (Applied Biosystem). Gene expression was examined using the StepOnePlus Real-Time PCR System and SYBR Green containing universal PCR master mix (Kapa SYBR Fast, Merck) in the presence of primers for the sequences listed below:

1. male fertility factor kl2
  *kl-2* FORWARD: GGAGTACCAGGAATGGTTCG
  *kl-2* REVERSE: TTGAATTAGGGCTCGAAAAAGC
2. Control for the experiment:
  *Drosocin*
  *Dro* FORWARD: ATTTGTCCACCACTCCAAGC
  *Dro* REVERSE: GATGACTTCTCCGCGGTATG
  *CecropinC*
  *CecC* FORWARD: CATCAGTCGCTCAGTTTCCA
  *CecC* REVERSE: TTCCCAGTCCTTGAATGGTT
3. Housekeeping gene:
  *Ribosomal protein L32*
  *RpL32* FORWARD: ATGCTAAGCTGTCGCACAAATG
  *RpL32* REVERSE: GTTCGATCCGTAACCGATGT

Amplification was performed by subjecting samples to 10 min of initial denaturation at 95 °C followed by three steps: 40 cycles of 15 s at 95 °C (denaturation), 30 s at 59 °C (annealing) and 30 s at 72 °C (extension). Product specificity was assessed by melting curve analysis, and selected samples were run on 1% agarose gels for size assessment. Knowing that the *kl-2* gene is expressed only in males, we used samples collected from females as an additional control for primers and we observed no amplification signal. A standard curve was used to calculate the gene expression level normalized to *RpL32*. 

### 2.8. Bacteria Load in Infected Flies

Seven-day-old males from the following crosses *act* > *male fertility factor kl2^RNAi^*, *act* > *Valium*, *w^1118^* > *male fertility factor kl2^RNAi^*, and *act* > *w^1118^* were infected as described above. To estimate the bacterial load 3 h after *E. coli* infection, flies from each treatment were individually crushed and homogenized using an electronic pestle in Eppendorf tubes containing 200 μL PBS. The number of bacteria (CFU) within each fly was estimated by serial dilution and plating on MHB agar plates. Plated bacteria were incubated at 37 °C. In total, 15 flies were used for each combination of fly strains/bacteria or PBS treatment, and the results are from 3 independent experiments. 

### 2.9. Transmission Electron Microscopy (TEM)

*E. coli*, *S. aureus* and *B. subtilis* were treated with chemerin-p4, MFF-p1 or PBS as control for 2 h at 37 °C. After the treatment, samples were washed three times in PBS and fixed overnight at 4 °C (*E. coli* in 2% glutaraldehyde in 0.1 M sodium cacodylate buffer; Gram-positive bacteria in 2.5% glutaraldehyde in PBS). *E. coli* was then post-fixed with 1% osmium tetroxide in 0.1 M cacodylic buffer at room temperature (RT) and stained *en bloc* with 2% uranyl acetate aqueous solution for 1 h at RT. *S. aureus* and *B. subtilis* were post-fixed with 1% osmium tetroxide for 2 h at 4 °C. After dehydration in a graded ethanol series (50–100%) and impregnation in propylene oxide, samples were embedded in epoxy resin (PolyBed 812, Polysciences, Inc., Warrington, PA, USA). Ultrathin sections (65 nm) were cut using an ultramicrotome (Leica EM UC7) and post stained with uranyl acetate and lead citrate. Specimens were observed using a transmission electron microscope (JEOL JEM 2100) operating at an accelerating voltage of 80 kV. 

### 2.10. Statistical Analysis

For statistical analysis, one-way ANOVA followed by Tukey’s or Sidak post hoc test or non-parametric Kruskal–Wallis ANOVA followed by the Dunn’s post hoc test or two-tailed Student’s t test were performed using the GraphPad Prism 9.1.2 software (La Jolla, CA, USA).

## 3. Results

### 3.1. Identification of Homologs of Chemerin-p4 Peptide

**Figure 1:**
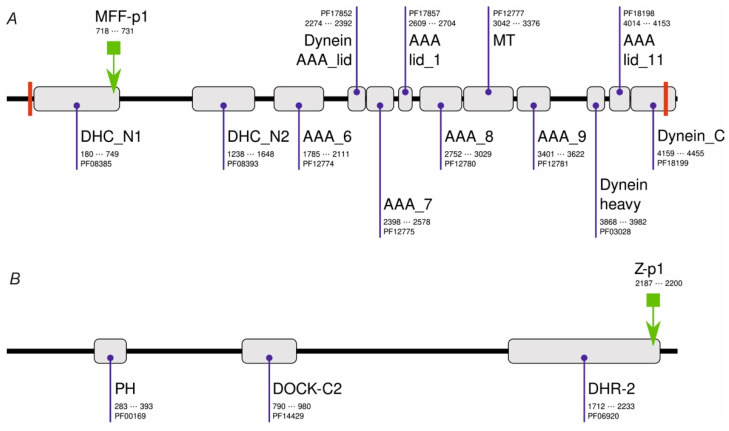
Domain architecture of MFF-kl2 (**A**) and Zizimin (**B**) of *Drosophila melanogaster*. PFAM domains are shown as gray boxes together with their names, PFAM accession codes, and alignment positions in protein sequences. Transmembrane helices are shown as red bars. The locations of MFF-p1 and Z-p1 are shown with the green arrow.

Initially, we performed PHI-BLAST scans of a non-redundant set of *Drosophila melanogaster* sequences using either chemerin-p4 or chemerin-8 (VR-15) [24] sequences as a query and the corresponding regular expression (RE) pattern (Table 1). These searches resulted in no hits. When the shortened RE patterns (Table 1) were used, several dozen hits were observed. Only one of them (MFF-p1, Table 1) had several features which were found previously to be prerequisites for the antibacterial activity of chemerin-p4-related peptides, i.e., at least 14 aa long, a net charge at least of +4, and the ability to adopt a strongly amphipathic structure (relative hydrophobic moment (rHM) > 0.375). MFF-p1 was identified in Male fertility factor kl2 (MFF-kl2, NCBI NR db id: NP_001015505.5, UniProtKB accession code: Q5LJP0_DROME) at the C-terminus of the DHC_N1 domain (Figure 1). MFF-kl2 is a 4459-aa protein involved in microtubule-based movement. The protein (Figure 1) comprises 12 domains mostly belonging to the dynein and AAA protein superfamilies, two transmembrane helices and several short natively disordered fragments. 

Another hit resulted in the alignment with a Zizimin fragment (NCBI NR db id: NP_001260200.1, UniProtKB accession code: M9PCG3_DROME). Zizimin is a 2298-amino-acid (aa) protein involved in compound eye development and signal transduction. It comprises four protein domains and numerous interspersed low-complexity regions. In contrast to MFF-p1, the Z-p1 peptide (Table 1) is only slightly cationic under neutral pH but similarly to the MFF-p1, the Z-p1 peptide shows marked amphipathicity when the α-helical conformation is assumed. 

**Figure 2:**
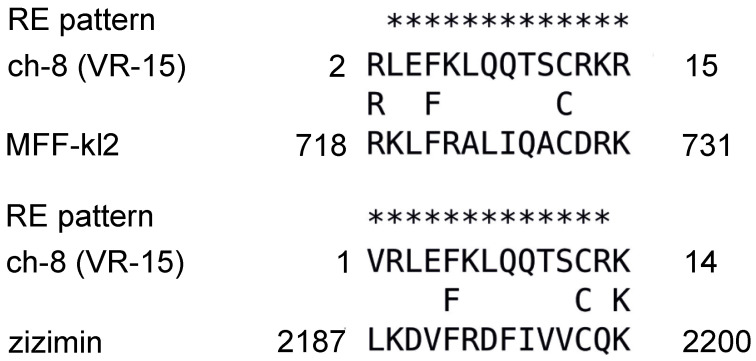
The PHI BLAST search results with the chemerin-8 (VR-15) peptide used as a query and MFF-kl2 (alignment score—3.4 bits; E-value—13; sequence identity—21%) and Zizimin (alignment score—3.1 bits; E-value—21; sequence identity—21%). Chemerin 8 (VR-15) is a 15-aa fragment of the human chemerin spanning from V66 to R80.

**Figure 3:**
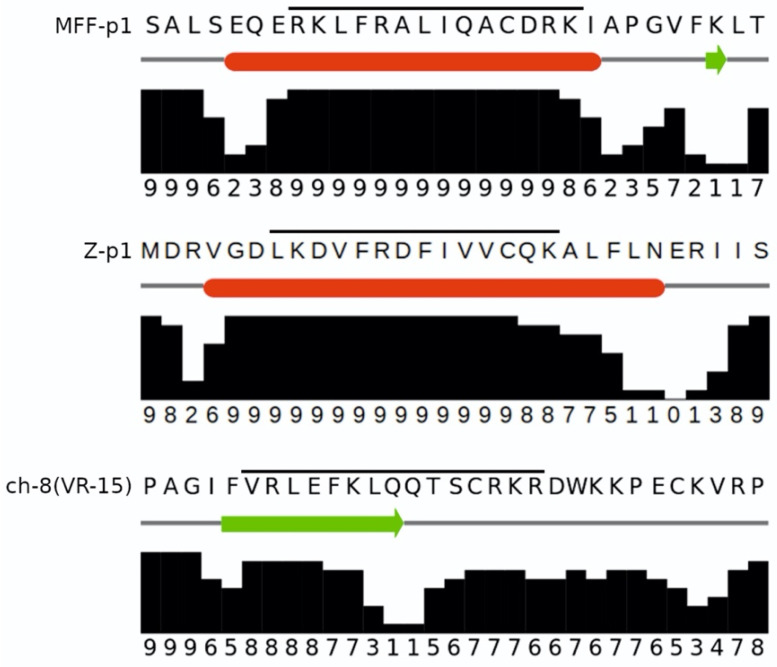
The JPRED4 secondary structure predictions for the MFF-p1 (**top**), Z-p1 (**middle**), and chemerin-8 (VR-15) (**bottom**) peptides. The fragment corresponding to the actual peptides is marked with a horizontal line. Predictions of an α-helix (red bars) and extended (β-strand, the green arrow) conformation are accompanied by JPRED4 confidence scores.

The PHI-BLAST alignments between chemerin-8 (VR-15) and both MFF-p1 and Z-p1 are shown in Figure 2. While there are no statistically significant signs of sequence similarity due to homology (low alignment scores and high E-values), the MFF-p1 peptide matches very well with the characteristics of the chemerin-p4 peptide in its fragment directly associated with antibacterial activity (i.e., 8 (VR-15)). It is worth noticing though that both MFF-p1 and Z-p1 manifest their amphipathicity not as an extended β-strand, as the chemerin-8 (VR-15) peptide does, but as an α-helix. To confirm the ability of MFF-p1 and Z-p1 peptides to adopt α-helical conformation, we used the JNET4 prediction server [28]. The results of the secondary structure predictions for MFF-p1 and chemerin-8 (VR-15) peptides are shown in Figure 3. Indeed, the predictions clearly indicate that both Drosophila peptides can preferentially form helices under the right conditions (e.g., in the anisotropic environment of lipid membranes) and both chemerin-p4 and chemerin-8 (VR-15) peptides are more likely to adopt an extended conformation. It is worth noting that the marked increase in rHMβ for the chemerin-8 (VR-15) peptide (Table 1) correlates well with the location of the β-strand predicted for this fragment of chemerin-p4 peptide (Figure 3, bottom).

### 3.2. Peptide MFF-p1 Demonstrates Antimicrobial Activity against E. coli and B. subtilis In Vitro

Our previous studies showed that mammalian chemerin-derived peptide 4 exhibits high antimicrobial potential. Among its antimicrobial targets are Gram-positive (*S. aureus*) and Gram-negative (*E. coli*) bacteria [16,23]. Furthermore, we have also demonstrated that MRSA strains have low or no resistance to chemerin-p4 [24]. The newly identified functional analogs of the chemerin-8 (VR-15) peptide, i.e., MFF-p1 and Z-p1, were tested against both Gram-negative and Gram-positive bacteria to determine if they retain the antimicrobial potential of the original chemerin-p4 peptide.

**Figure 4:**
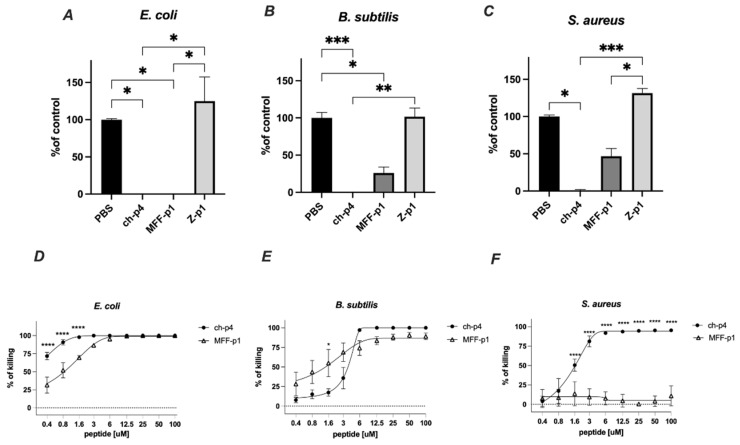
MFF-p1 is bactericidal in vitro. (**A**–**C**): *E. coli* (HB101 strain), *B. subtilis* (ATCC 9372 strain), and *S. aureus* (8325-4 strain) were incubated with chemerin-p4, MFF-p1 and Z-p1 (100 μM) peptides for 2 h. Cell viability was analyzed by MDA assay. N = 3, mean ± SEM, ***, and *p* < 0.001; **, *p* < 0.01; and *, *p* < 0.05 by Kruskal–Wallis one-way ANOVA with post hoc Dunn’s test. (**D**–**F**): Bacteria were incubated with chemerin-p4 or MFF-p1 peptides at the indicated concentrations for 2 h. Data show the percentage of killing (0%—control group). Mean ± SEM of three independent measurements is shown. ****, *p* < 0.0001; *, *p* < 0.05 by one-way ANOVA with post hoc Sidak test.

The selected peptides (100 μM) were tested for antimicrobial activity against *E. coli* HB101, *S. aureus* 8325-4 and *B. subtilis* ATCC 9387 strains using an MDA assay. Among them, MFF-p1 exhibited the strongest antimicrobial potency, resulting in the almost complete inhibition of viable counts of *E. coli* and *B. subtilis* following 2 h of the treatment. In the case of *S. aureus*, the inhibitory effect of MFF-p1 was less robust than that of *E. coli* or *B. subtilis*. Peptide Z-p1 did not demonstrate antimicrobial activity against the selected bacteria (Figure 4). 

The antimicrobial activity of MFF-p1 was further confirmed through the determination of minimal inhibitory concentration (MIC) values. We previously showed that *E. coli* exhibits a high sensitivity to the mammalian chemerin-p4, with an MIC value between 6.3 and 12.5 μm [24]. Herein, we demonstrate that both a concentration-dependent survival curve and MIC in the range of 6–12.5 μM (Figure 4D) in MFF-p1-treated *E. coli* are very similar to chemerin-p4 [24]. In contrast to *E. coli*, the antimicrobial activity of MFF-p1 against Gram-positive bacteria differed from chemerin-p4. *B. subtilis* treated with chemerin-p4 had a similar MIC to *E. coli*, while MFF-p1 significantly lowered the survival rate, but even at a high concentration (100 μM) the percent of killing was around 90%, not 100% (Figure 4B,E). In contrast to chemerin-p4, *S. aureus* seems to be resistant to MFF-p1 antimicrobial challenge (Figure 4C,F).

### 3.3. MFF-p1 Disrupts Integrity of Bacterial Superficial Layers

**Figure 5:**
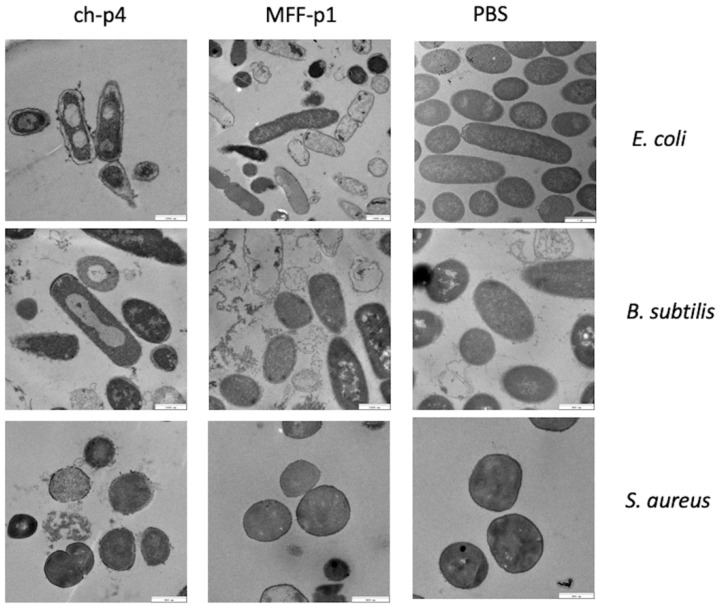
MFF-p1 disrupts the integrity of the superficial layers of bacteria. *E. coli* (HB101 strain)*, B. subtilis* (ATCC 9372 strain), and *S. aureus* (8325-4 strain) were incubated with chemerin-p4 (ch-p4) and MFF-p1 (100 μM) peptides for 2 h. Bacteria morphology was assessed by transmission electron microscopy. The images in each panel are from one experiment and are representative of at least three independent experiments.

We have already demonstrated that chemerin-p4 affects bacteria morphology even after 5 min of treatment [24]. In another previous study, we showed, using scanning and transmission electron microscopy, extensive damage to the structure of the bacteria cell surface in Gram-positive and Gram-negative bacteria [29]. This indicates a disruption of bacterial membranes and peptidoglycan layers. Analyses of the results from transmission electron microscopy in the present study also showed massive changes in the cell envelope and bacteria morphology, both in *E. coli* and *B. subtilis* strains treated with MFF-p1. These changes resembled those made by chemerin-p4 in the previous study. In contrast to these results, no changes in morphology were observed in *S. aureus* after treatment with MFF-p1. These data further suggest that *S. aureus* is quite resistant to MFF-p1 (Figure 5). 

### 3.4. The Expression of Male Fertility Factor kl-2 Is Clock Controlled

**Figure 6:**
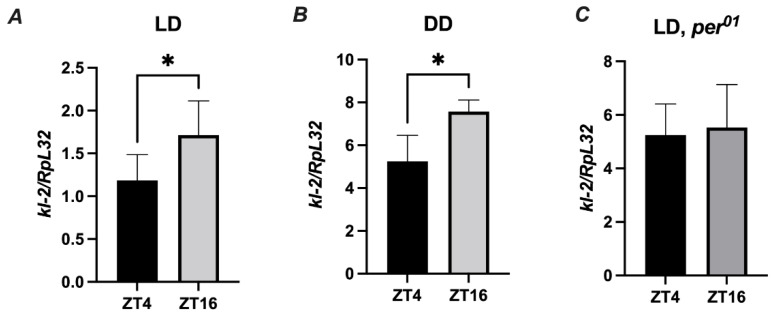
*kl-2* expression in *D. melanogaster* is rhythmic. *Canton S* (**A**,**B**) and *per^01^* flies were maintained in light/dark LD 12:12 (**A**,**C**) or constant darkness conditions (**B**). Flies were collected at ZT4 (four hours from the beginning of the day) or ZT16 (four hours from the beginning of the night) and total RNA from the abdomen was subjected to RT-qPCR. Relative expression of *kl-2* is shown as mean ± SD (N = 5 independent experiments, with 25 flies in each). Statistically significant differences between groups are indicated as * *p* < 0.05 by two-tailed Student’s *t*-test.

Since *Drosophila* immunity and behavior is regulated by the circadian clock, it is important to perform experiments at the same time of the day across experimental replicates [30]. To evaluate the role of the MFF-kl2 protein in antibacterial defense at a specific time of the day, we investigated whether the *kl-2* gene is expressed in *D. melanogaster* in a rhythmic manner. We found a statistically significant difference in the expression levels of *kl-2* between two time points ZT4 (four hours after lights on) and ZT16 (four hours after lights off) under light/dark LD12:12 conditions in the abdomen of wild-type (Canton S) flies (Figure 6A). The same pattern of rhythm was observed in flies under constant darkness (DD) conditions (Figure 6B). In the clock, for mutants of *per^01^*, the level of kl-2 mRNA was similar at all time points studied (Figure 6C). These results suggest that *kl-2* expression is controlled by the circadian clock. 

### 3.5. Male Fertility Factor kl2 Is Upregulated by Bacteria and Controls Bacteria Load in D. melanogaster

**Figure 7:**
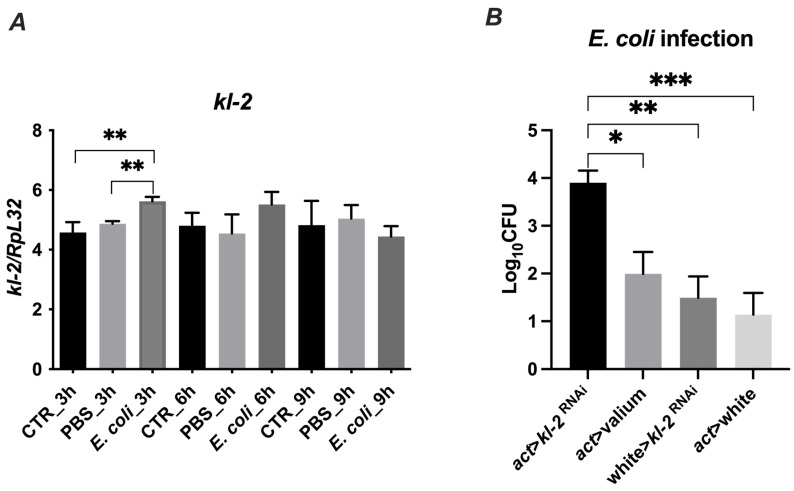
Gene expression (**A**) and antibacterial protection of MFF kl2 (**B**) in response to microbial challenge. (**A**) Bacteria upregulate the mRNA expression of *kl-2*. Canton S flies were infected by injection with *E. coli* (*E. coli* group), injected with PBS (PBS group) or untreated (CTR group) and collected 3 h, 6 h and 9 h after infection, and total RNA was subjected to RT-qPCR. Relative expression of *kl-2* is shown as mean ± SD (N = 3 independent experiments, with 15 flies in each). Statistically significant differences between groups are indicated as ** *p* < 0.01 by two-tailed Student’s *t*-test. (**B**) Male fertility factor kl2 is bactericidal in vivo. The experimental flies (*act* > *kl-2^RNAi^*) and controls (*act* > *Valium*, *w^118^* > *kl-2^RNAi^*, *act* > *w^1118^*) were infected with *E. coli*. Three hours after infection, flies were collected and bacterial load was determined by colony-forming assay. The results are shown as means ± SEM. Statistically significant differences between groups are indicated as ***, *p* < 0.001; **, *p* < 0.01; and *, *p* < 0.05 by Kruskal–Wallis one-way ANOVA with post hoc Dunn’s test.

Next, we investigated whether the *kl-2* gene expression in *D. melanogaster* is modulated by exposure to bacteria. We used the *E. coli* bacteria strain, which is susceptible to MFF-p1-dependent killing, and observed a significant upregulation of *kl-2* gene expression in infected flies after 3 h of the treatment (Figure 7A). This upregulation corresponded with the upregulated mRNA levels of the well-known antimicrobial proteins of *Drosophila*—Drosocin and Cecropin C (Figure S1).

To determine whether the MFF-kl2 protein plays a role in controlling bacterial burden in flies, we used transgenic flies with the silenced expression of the *kl-2* gene encoding MFF-kl2 under an actin promotor (*act* > *kl-2^RNAi^*) and adequate controls, which are progeny from crosses of parental lines used to make experimental flies with control lines [*act* > *Valium* (*Valium*—the line with *VALIUM* vector, the control for RNAi lines), *w^118^* > *kl-2^RNAi^*, *act* > *w^1118^* (*w^118^*—null mutation of the white gene encoding ABC transporter, which was used as control because of the genetic background of both the *actin* driver line (*act*) and *kl-2^RNAi^* line)]. The experimental and control flies were infected with *E. coli* and the bacterial load was recovered from the flies 3 h later and analyzed by a colony-forming assay. The kl-2-deficient flies possessed at least two-fold higher bacteria burden compared to control lines (Figure 7B). These data suggest that the MFF-kl2 protein comprising the MFF-p1 peptide plays an important role in restricting bacterial infection in *D. melanogaster*.

## 4. Discussion

*D. melanogaster* is an important model species for studying the evolution of animal immunity and in therapeutic drug discovery, thanks to similarities in highly conserved innate immune pathways between the fruit fly and mammals [31,32]. These include two NF-κB signaling pathways, as well as Toll and immune deficiency (IMD) pathways, which are similar to mammalian TLR pathways [33]. Both pathways are responsible for the activation of genes encoding AMPs in fruit flies [32,34]. Usually, flies share approximately 40% of their protein sequence identity with their mammalian homologs, although similarity can be greater than 90% in highly conserved domains [32]. Recently, we showed that human chemerin and its antimicrobial derivatives support the barrier function of skin and oral cavity epithelia [23,24,29]. Based on these findings, we used NCBI PHI BLAST to search the NR Protein database to identify peptides from *D. melanogaster*, matching the characteristics of the antimicrobial chemerin-p4 peptide. We found two such peptides: MFF-p1 (coded by *kl-2* gene) and Z-p1 (coded by *Zizimin*). Although chemerin has no orthologs in invertebrates [18] and both predicted peptides have no statistically significant signs of sequence similarity to chemerin-p4, the MFF-p1 peptide possesses several features that correspond to the characteristics of the most bactericidal fragment of chemerin-p4, i.e., chemerin-8 (VR-15) [24]. In contrast to Z-p1, which seems to lack antimicrobial potential, MFF-p1 exhibits bactericidal activity against *E. coli* and *B. subtilis*, probably due to the features that include a positive net charge (+4) and ability to adopt an amphipathic structure. The antimicrobial effect of MFF-p1 against *S. aureus* was likely limited by differences in the structure and synthesis of the cell walls of Gram-positive bacteria, such as *S. aureus* and *B. subtilis*, which may affect their susceptibility to AMPs [35,36]. In another view, based on the disparities in IMD and Toll-signaling pathways, IMD-responsive AMP genes are essential for combating Gram-negative bacteria, whereas Toll-regulated AMPs contribute rather to resistance to fungi and Gram-positive bacteria [8,37]. Nevertheless, for the model of antimicrobial action of MFF-p1, as well as the signaling pathway responsible for upregulation, *kl-2* remains uncharacterized. 

The dimorphism between males and females of *D. melanogaster* differentiates their sensitivity to pathogens [34]. Many genes located on the X chromosome are related to immune responses, including those involved in the signal transduction via IMD and Toll pathways that control AMPs synthesis and secretion [34,38]. Since the Y chromosome carries genes associated mostly with male reproduction, there is growing evidence of the involvement of the Y chromosome in shaping the immunity of *Drosophila* males [34,39]. It was previously shown that multiple chromosome Y-encoded genes (*Pp1-Y1*, *kl-5*, *kl-3*, *Ppr-Y*, *CCY*, and *FDY*) are involved in antimicrobial responses in *Drosophila* [34]. The fertility factors *kl-2*, *kl-3*, and *kl-5* encode three different axonemal dynein heavy-chain polypeptides, which are a part of the flagellar motor complex [34,40,41]. Mutations in these fertility factors cause male sterility [34,40]. Moreover, the disruption of two of them, *kl-3* and *kl-5*, makes flies more susceptible to *Serratia liquefaciens* infection [34]. However, the role of *kl-2* in the antimicrobial response has not been shown yet. In our experiments, we observed that, in the *kl-2* gene-deficient flies, at least two-fold more bacteria persisted after *E. coli* infection compared to the control. Additionally, we found an increased expression of the *kl-2* gene in infected flies. Notably, the upregulation of *kl-2* coincides with the increased expression of other AMPs, i.e., *Drosocin* and *Cecropin*, whose inducible expression in epithelia is controlled by IMD signaling [42,43]. In conclusion, the obtained results indicate that the MFF-kl2 protein protects *D. melanogaster* against bacteria. We propose a mechanism in which MFF-kl2 may directly control the microbial burden through the antibacterial properties of its antimicrobial peptide, MFF-p1. This peptide is similar in the sequence of amino acids to the human chemerin-derived peptide p4. Further understanding of the antimicrobial mechanisms of MFF-p1 and MFF-kl2 activity, and more importantly how *kl-2* expression is regulated during microbial infection (including the involvement of IMD and/or Toll-signaling pathways), may help in expanding our knowledge on undiscovered factors in innate immune responses in fruit flies.

## Figures and Tables

**Table 1:** Sequence and selected properties of peptides; chemerin-p4 (ch-p4), chemerin-8 (ch-8) (VR-15), MFF-p1 and Z-p1. For each peptide, the amino acid sequence and corresponding RE pattern, amino acid length (#aa), and net charge (at neutral pH) are given. rHMβ and rHMα are relative hydrophobic moments calculated assuming the fully extended (β-strand) and α-helical conformation, respectively.

Peptide Name	Peptide Sequence RE Pattern	#aa	Net Charge	rHMβ	rHMα
ch-p4	VRLEFKLQQTSCRKRDWKKP	20	+5	0.375	0.157
	[LIV]-[KRH]-X(2)-[WYF]-[KRH]-X(5)-C-X-[KRH](2)-X-[WYF]-[KRH]-X(2)				
ch-8 (VR-15)	VRLEFKLQQTSCRKR	15	+4	0.625	0.108
	[LIV]-[KRH]-X(2)-[WYF]-[KRH]-X(5)-C-X-[KRH]-[KRH]				
MFF-p1	RKLFRALIQACDRK	14	+4	0.143	0.548
	X(2)-[WYF]-[KRH]-X(5)-C-X-[KRH](2)				
Z-p1	LKDVFRDFIVVCQK	14	+1	0.152	0.618
	[LIV]-[KRH]-X(2)-[WYF]-[KRH]-X(5)-C-X				

## Data Availability

The data presented in this study are available on request from the corresponding author.

## Supplementary Materials

The following are available online at https://www.mdpi.com/article/10.3390/cimb44030076/s1. Figure S1: Up-regulation of AMPs- encoding genes after infection – control.
