# Supplementary material for: Antimicrobial Properties of a Peptide Derived from the Male Fertility Factor kl2 Protein of Drosophila melanogaster
Source: Curr Issues Mol Biol. 2022 Feb 28;44(3):1169–81. doi: 10.3390/cimb44030076 (PMC8947439; doi:10.3390/cimb44030076)
Supplement: Supplementary file 1 [file cimb-44-00076-s001.zip › cimb-1551396-SI.pdf]

Supporting information:

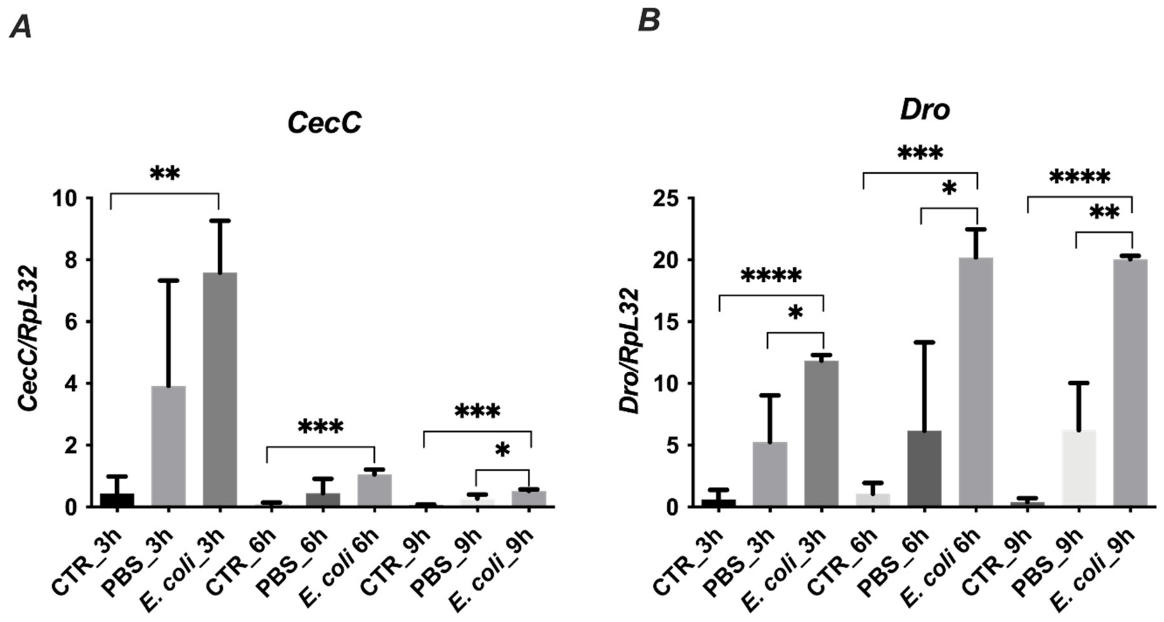

Figure S1. <u>Up-regulation of AMPs- encoding genes ((**A**) – *Cecropin C* ; (**B**) – *Drosocin*) after infection – control.</u> Canton S flies were infected with *E. coli*, 3 h, 6 h and 9 h after infection flies were collected and total RNA was subjected to RT-qPCR. Relative expression of indicated genes are shown as a mean ± SD (n = 3 independent experiments, with 15 flies in each). Statistically significant differences between groups are indicated as **** $p< 0.0001$; *** $p < 0.001$; ** $p < 0.01$; * $p < 0.05$ by two tailed Students *t*-test.
